# Management of Mass Death in COVID-19 Pandemic in an Indian Perspective

**DOI:** 10.1017/dmp.2020.399

**Published:** 2020-10-22

**Authors:** Rajanikanta Swain, Jyotiranajan Sahoo, Sudhanshu P. Biswal, Asit K. Sikary

**Affiliations:** 1 Department of Forensic Medicine, Kalinga Institute of Medical Sciences, Bhubaneshwar, Odisha, India; 2 Department of Community Medicine, Institute of Medical Sciences & SUM Hospital, Shiksha ‘o’ Anusandhan Deemed to be University, Bhubaneshwar, Odisha, India; 3 Department of Microbiology, SCB Medical College, Cuttack, Odisha, India; 4 Department of Forensic Medicine, ESIC Medical College & Hospital, Faridabad, India

**Keywords:** guidelines, health resources, infectious disease, mass disaster, public health

## Abstract

Current international experience has shown the vulnerability of health-care systems of developed nations, and of developing nations such as India, to coronavirus disease 2019 (COVID-19). COVID-19 pandemic is a disaster with mass casualties. International experience has revealed that, even in the countries where mass disasters are less frequent and not involved in conflicts, they are overwhelmed with COVID-19 deaths. Although, in the current scenario with fewer deaths, India’s health-care system can handle the situation of COVID-19 but should be prepared for the worst in terms of appropriate management, and adequate infection prevention measures including handling the dead without hampering the dignity of the deceased and of the surviving family. Before any crisis overwhelms responders and resources, emergency response plans should be established and activated to ensure the reliable identification and documentation of the dead. The current review was carried out to recommend the proper management of dead bodies in the COVID-19 mass disaster with a particular focus on resource-poor countries, such as India.

Coronavirus disease 2019 (COVID-19) is caused by severe acute respiratory syndrome coronavirus 2 (SARS-CoV-2), classified under hazard group-3 (HG3) pathogen by the Advisory Committee on Dangerous Pathogens (ACDP).^1^ Person-to-person transmission of SARS-CoV-2/COVID-19 occurs by means of direct contact or through virus-laden droplets generated through coughing and sneezing of the infected person.^2^ As of now, long-distance transmission through the air is unlikely.^3^ The virus is highly stable on different surfaces, such as 4 days on banknotes and glass surfaces, and 7 days on stainless steel, plastic, and the outer surface of the surgical mask. Although the virus is highly infectious at 4°C, it loses its infectivity within 5 min at 70°C.^4^ The infectivity is still unclear in human tissue after the death of the host.^5^

Regarding the case fatality rate of COVID-19, although mortality is higher among elderly individuals with co-morbid conditions, it can kill healthy adults too. Available literature suggests a case fatality rate of around 1%, but even at this low rate, COVID-19 is more severe than the common influenza pandemic of the early 1900s.^6^ Moreover, the synergistic effect of transmissibility and the case fatality rate can make COVID-19 a once-in-a-century pandemic with the highest number of deaths reported. Health systems are trying hard to increase the survival rate, but death seems inevitable at certain instances. Health systems, worldwide, may not be able to handle such a high death rate in a short time, and this situation may be compounded in resource-poor countries, such as India.

Current evidence suggests no risk of transmission of infection from a dead body infected with COVID-19.^7^ However, the natural history of the COVID-19 is unknown and unfolds with time, creating doubts on such claims. The current pandemic of COVID-19 has turned into a mass disaster situation of biological origin. The morgues are overwhelmed with the sudden rise in the number of dead bodies without any disposal plan in place.^8,9^ Near and dear ones of those who are deceased are unwilling to handle dead bodies due to the inherent fear of contracting the disease. Such circumstances can make the current pandemic like any natural environmental disaster where the dead bodies are buried in mass graves with little record of who died, how they died, and where the bodies were disposed of. Moreover, failure to maintain a proper record can also hinder the contact tracing of COVID-19. In addition, not knowing the place of disposal could be further devastating for emotionally suffering family members of the decedents.

COVID-19 pandemic is a disaster with mass casualties.^10^ Even those countries, where mass disasters are less frequent or that are not involved in conflicts, are overwhelmed with COVID-19 deaths. Before any crisis overwhelms responders and resources, emergency response plans should be established and activated to institute proper sanitation measures while handling the dead bodies for storage, preprocessing before handing them over to transport facility, and for their cremation or burial. The process for obtaining death certificates, death registration, and burial permits should be facilitated. Such preparedness will help to avoid undignified death management.

The current review was carried out to recommend the proper management of dead bodies, including recommendations on prevention of infection, respect of religious beliefs of the deceased, storage and transport of the dead bodies, and management of resources, in a possible COVID-19 mass disaster scenario in resource-poor countries, such as India.

## Covid-19 is a Public Health Emergency

COVID-19 started as an outbreak in Wuhan city, China, and continues to spread worldwide.^11^ The World Health Organization (WHO) decided to define the outbreak of COVID-19 as a public health emergency of international concern on January 7, 2020, which triggered the release of funding and other resources.^12^

Two main reasons that make COVID-19 a threat to the public are the efficient transmission of the virus and the case fatality rate. In statistical terms, the transmissibility of infection is represented by a reproductive number (R_0_). The R_0_ is quite higher for SARS-CoV-2 in comparison to SARS coronavirus, with an average of 3.28 (range, 1.95 to 6.47).^13^ At this rate, there should be an exponential rise in cases than what the world is now experiencing, if not controlled.^14^ Evidence also suggests it can be transmitted from mild symptomatic or asymptomatic cases, making it harder to control than the SARS pandemic in 2002-03 and the Middle East respiratory syndrome (MERS) in 2012-2013.^15^ As of August 16, 2020, a total of 216 countries have been affected, with more than 21,295,845 confirmed cases and 761,779 deaths due to COVID-19 worldwide.^16^ The case fatality rate at this time was calculated to be ~6.3%; a roughly 3-fold jump from 2.2% on January 30, 2020.^2,11^ These numbers, however, may be an underestimation of the cases due to limited testing.

## Current Recommendation by the WHO for Management of Covid-19 Dead Bodies

Dead bodies are generally noninfectious except in category-4 diseases, such as viral hemorrhagic fever like Ebola, Hanta, and category-3 diseases like hepatitis-B (HBV), acquired immunodeficiency syndrome (AIDS), tuberculosis (TB), and rabies.^17^ However, if the lung tissue of COVID-19 deceased persons is handled improperly during the autopsy, it can be infectious.^7^ As SARS-CoV-2 causing COVID-19 pandemic disease is a new virus whose virulence and duration of survival in the dead body is not yet entirely understood, more precautions may be taken to prevent further spread during handling of dead bodies.

National and international organizations/governments have been formulating different guidelines about the management of dead bodies according to the local context. According to the WHO, these cases should be managed on a case-by-case basis. It should be a balance between the rights of the family to perform the last rites and the risk of exposure to infection. The current guidelines say^3,7^: Children, geriatrics of more than 60 years of age, and the immunocompromised person should avoid coming in contact with the deceased having COVID-19 infection; embalming and autopsy are not recommended; closing off all the natural orifices and iatrogenic wounds of the dead bodies with cotton balls soaked with 1% hypochlorite while shifting the dead bodies from the hospital; packing of the body in a puncture-proof body bag with sensitization; cremation is not necessary; it is a matter of cultural choice and available resources; hand hygiene, social distancing, and contact precaution are always required during the whole process; and, ensure that the hospital personnel who are going to deal with the dead body should be equipped with appropriate PPE, including face shield and goggles.

These guidelines are meant for all the countries worldwide. Developed countries with their ample resources can easily follow it. On the other hand, in resource-poor countries usually untrained or minimally trained hospital staff or morgue staff handles dead bodies. Hence, proper training of these staff in dealing with the COVID-19 dead bodies should be the first priority. Additionally, adequate supplies of personal protective equipment (PPE) for body handlers and puncture-proof body bags for packaging and transportation of dead bodies are also a challenge in resource-poor countries, especially in a possible scenario of mass disaster.

## Guidelines are Needed for Management of Covid-19 Mass Fatalities in Indian Context

Current international experience shows the vulnerability of the health-care system of developed nations, and of developing nations, such as India, to COVID-19. India’s health-care systems can handle the current COVID-19 situation because of the lower burden of deaths, but they should prepare for higher case fatality rates by instituting appropriate management of the dead and adequate infection prevention measures that are sensitive to the dignity of the deceased persons and their surviving family. Recommendations or guidelines should be available to follow during mass disaster scenarios. Considering the limited resources within the country, the recommendations or guidelines should consider the following areas:

### Prevention of Infection

The 2 most essential things before disposal are the identification of the body and control/prevention of the disease.^18^ As the identification of dead bodies due to COVID-19 is mostly well established (as the body is not disfigured, death at the hospital is recorded and a next of kin is usually available to identify the body in the event of death at home), prevention and control of infection remain the most critical step. In this respect, the following items are suggested: maintenance of personal hygiene (including hand hygiene, proper donning of PPE before handling of the dead body, avoidance of sharps injury, and proper doffing after handling of the dead body) by all persons involved in the direct or indirect handling of dead bodies,^19^ disinfection of the dead body with chlorine-based solution, keeping of the body in a puncture-proof body bag,^5,20^ minimal direct contact with the body, proper use of PPE by individuals who come in contact with the decedent whether they are in the hospital or outside the hospital, and cremation or burial in airtight boxes.^3,18^ However, availability of trained staff for dead body handling and availability of logistics, such as PPE kit, chemicals used for sanitization measures, and puncture-proof bags for dead bodies, are the major concerns in resource-poor countries.

### Respecting Religious Beliefs

With regard to religious beliefs, there are 2 methods of disposal of bodies, ie, either cremation or burial. Logically, cremation is a better method in COVID-19 deaths regarding its infectious nature. However, international agencies raised concerns over the cremation of the COVID-19 dead body outside the religious beliefs as it can cause social unrest.^10^ India is a country with various religions and should adopt both the methods formulating proper guidelines for each.

### Storage and Transport of Dead Bodies

Worldwide, countries facing mass deaths were overwhelmed with the numbers, and their morgue and cemeteries ran out of room.^21^ Moreover, the storage of dead bodies in the morgue is not the best practice in COVID-19 death, as infection can spread with unnecessary handling of the bodies, and identification/claims is not a significant issue. Immediate transportation to the cremation or burial sites will prevent the overwhelming of the morgue storage capacity, especially in resource-poor countries, which have limited morgue capacity to begin with. Therefore, around the clock transportation of dead bodies and identifying additional burial or cremation sites is key along with proper communication and coordination. Designated sites for cremation and burial are of utmost importance to avoid movement of dead bodies from one place to another. During the transit period, ie, from death to cremation, packing of the dead body in disinfected body bags is key to prevent infection, as suggested by the WHO, Ministry of Health and Family Welfare of the Government of India, and other international agencies.^7,20,22^

### Management of Resources

The worrying scenario is overwhelming demand on limited human and logistics resources, as pointed out by the Public Health Agency of Canada and the WHO.^23,24^ The resources should be properly mobilized to ensure the dignity of deceased persons, respect for the bereaved, and the identification and traceability of deceased persons. In this respect, the Canadian Government has put forth a detailed guideline that can be used in mobilizing resources and in using alterntives.^23^ The authors recommend adopting the same guidelines with some modifications, such as all relevant health-care workers to be trained in body handling, including senitization and packaging, transport facility (stretcher, trolley, etc.) to be earmarked for the dead bodies within the hospital premises, the capacity of cold storage to be increased along with arrangement of temporary refrigerated cabinets such as refrigerated trucks, unnecessary autopsy on COVID-19 death cases to be avoided, alternate transport facility (other than hearse van) to be identified to transport the bodies to the cremation/burial ground, and additional staff to be trained for cremation or burial of the bodies. Identification of bulk suppliers of logistics, such as body bags, mortuary sheets, PPE kits, and sanitizers, is important as well.

## Conclusions

COVID-19 is a threat to the whole world. Its transmission, not only from symptomatic patients but also asymptomatic individuals, makes it difficult to prevent the spread of disease. Although the mortality is higher among elderly individuals with a case fatality rate of around 18%, the rate may be higher in a populous country such as India due to the synergistic effect of its transmissibility and case fatality. Even though we are in the midst of the crisis, plans must be created to deal with the present as well as future situations, if the condition worsens and overwhelms the capacity of the health-care system. Even if there is no evidence of the spread of COVID-19 disease from the deceased person, the natural history of the disease continues to unfold and remains unknown. Hence, we must take due care while handling the dead bodies.
